# Benign maculopapular rash following dengue vaccination with Butantan-DV: a case series^؆^

**DOI:** 10.1016/j.abd.2026.501392

**Published:** 2026-06-16

**Authors:** Thays Herbst Carvalho, Aluísio José de Oliveira Monteiro Neto, Izabelle Ferreira da Silva Mazeto Bonini, Marianna Zaffani Machado de Oliveira, Marília Fomentini Scotton Jorge, Hélio Amante Miot

**Affiliations:** Department of Infectology, Dermatology, Imaging Diagnosis and Radiotherapy, Faculty of Medicine, Universidade Estadual Paulista, Botucatu, SP, Brazil

*Dear Editor,*

Dengue remains a major public health concern in Brazil and other tropical countries, with a significant impact on morbidity and healthcare burden. Vaccination has been incorporated as a complementary measure to vector-control programs. In this context, the new single-dose live-attenuated tetravalent dengue vaccine developed by the Butantan Institute (Butantan-DV) represents an important advance in dengue prevention.^1^, ^2^

Phase 3 clinical trials involving more than 16,000 participants demonstrated high efficacy and a favorable safety profile for Butantan-DV. Most adverse events were classified as mild to moderate, while serious vaccine-related adverse events were rare (<0.1%) and all resolved without sequelae. Among the most frequently reported adverse reactions within the first 21-days post-vaccination were injection-site pain/edema, headache, fatigue, myalgia, nausea, arthralgia, low-grade fever, and photophobia, in addition to dermatologic manifestations such as rash and pruritus. The incidence of rash was 22.5% among vaccine recipients, compared with 4.2% in the placebo group within the first 21 days after vaccination.^2^, ^3^, ^4^

Post-vaccination cutaneous reactions, particularly delayed eruptions such as maculopapular rash and urticaria, have been described following different vaccine platforms and are generally benign.^5^ Their clinical characterization is essential for appropriate dermatologic recognition and management, as well as for patient counseling.

Botucatu (SP), Maranguape (CE), and Nova Lima (MG) were the first municipalities to implement large-scale vaccination with Butantan-DV as part of a Brazilian Ministry of Health pilot program. Data on post-vaccination cutaneous manifestations in real-world settings remain scarce.

We report a case series of 11 patients from Botucatu (SP), Brazil, who developed a benign, delayed-onset, erythematous cutaneous rash after a single dose of the Butantan-DV dengue vaccine, focusing on the morphological characterization of skin lesions and associated systemic symptoms. Table 1 summarizes the main characteristics of the patients. In all cases, the cutaneous eruption was maculopapular (Fig. 1, Fig. 2), predominantly involving the trunk and proximal extremities. Dermatoscopically (Fig. 3), the papules displayed a blanchable, structureless erythematous background with scattered monomorphic dotted vessels, without purpuric globules or vasculitic features.

**Table 1:** Main demographic, clinical, and laboratory characteristics of the series of patients with skin eruption following Butantan-DV vaccination.

Age/Sex	Rash onset (days from vaccination)	Associated symptoms	Lymphadenopathy	Hematocrit (%)	Platelets (10^3^/mL)	Tourniquet test	Prior dengue
27/F	9	Diarrhea, nausea, pruritus	No	37.2	247,000	Negative	No
28/F	8	None	Yes	41.6	254,000	Negative	No
35/M	7	Headache	No	41.2	207,000	Positive	Yes
36/F	9	None	No	38.0	158,000	Negative	No
59/F	10	Diarrhea, nausea	No	46.0	209,000	Positive	No
57/F	9	Headache, myalgia, pruritus	No	40.5	347,000	Negative	No
27/F	6	Headache	No	40.3	304,000	Negative	No
31/F	9	None	No	40.0	289,000	Positive	No
57/F	10	None	No	45.0	186,000	Negative	No
32/M	9	Myalgia	No	‒	‒	Negative	No
27/F	13	Headache, pruritus	Yes	‒	‒	Negative	No

**Fig. 1:**
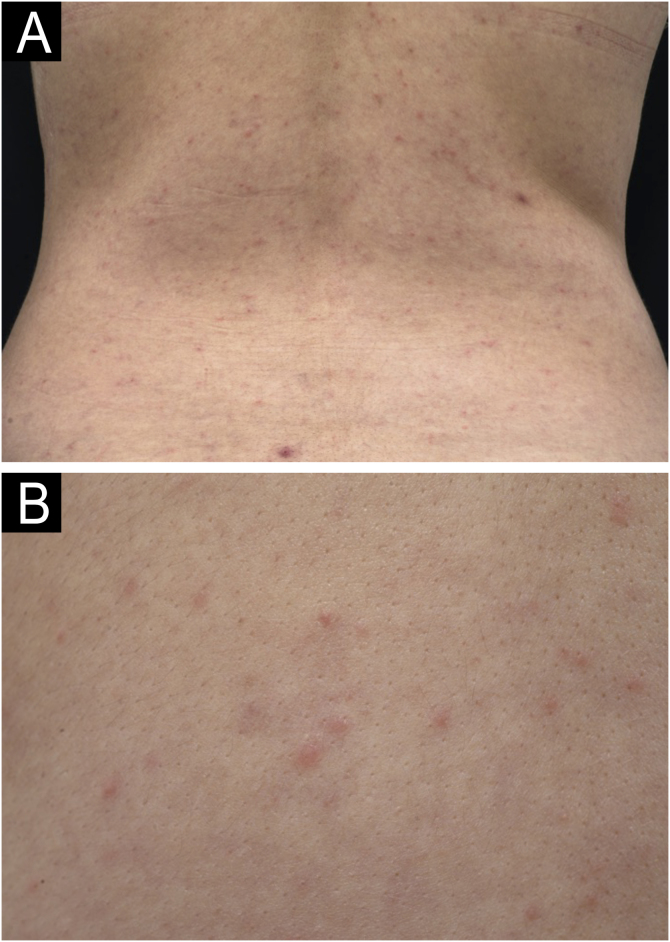
Maculopapular rash following dengue vaccination with Butantan-DV. (A) Erythematous papules and macules on the lumbar region. (B) Close-up of the non-follicular papules and macules on the abdomen.

**Fig. 2:**
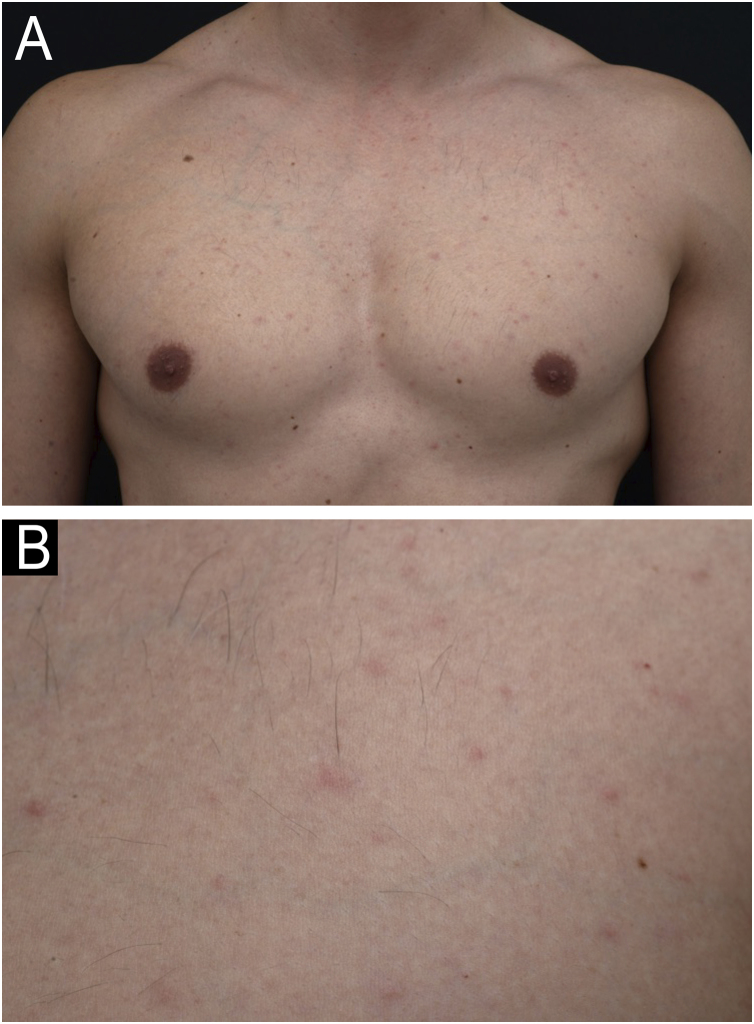
Maculopapular rash following dengue vaccination with Butantan-DV. (A) Erythematous papules and macules on the chest, epigastrium, and upper arms. (B) Close-up of the non-follicular papules and macules on the chest.

**Fig. 3:**
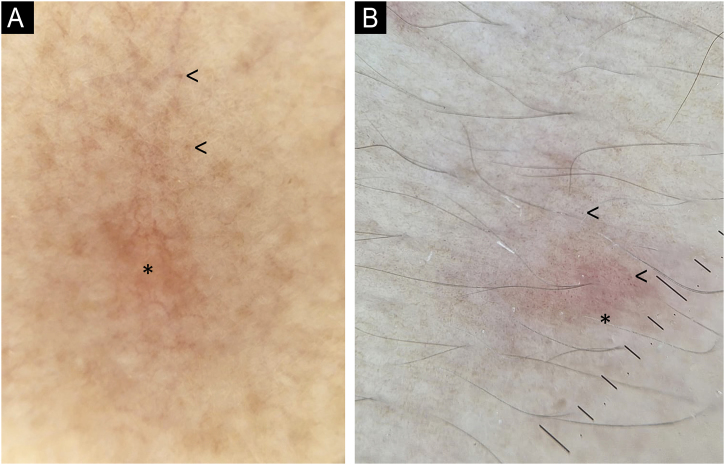
(A and B) Dermoscopy of papules from the maculopapular rash following dengue vaccination with Butantan-DV. (*) Indicates blanchable erythema. (<) Indicates scattered dotted vessels.

Lesions appeared 6‒13 days post-vaccination, with a centrifugal progression pattern. Mild pruritus was observed in three patients (27%). Lymphadenopathy was identified in two cases (18%). Three patients (27%) had a positive tourniquet test, and only one patient (9%) reported a prior history of dengue infection. No abnormalities in hematocrit values or platelet counts were observed. Mild systemic symptoms were present in seven patients (64%), most commonly headache, nausea, diarrhea, and myalgia. The duration of cutaneous lesions was approximately five days, with complete resolution in all cases. No residual desquamation or post-inflammatory hyperpigmentation was observed. No patient required medical therapy or hospital admission.

Delayed cutaneous reactions, including maculopapular rash and urticaria, are among the most commonly reported adverse events following vaccination.^5^ They have been described after live-attenuated, inactivated, and more recently developed vaccine platforms. In most cases, these manifestations are self-limited and do not contraindicate subsequent vaccine doses. In addition to the antigenic component, vaccines contain adjuvants and residual manufacturing elements that may modulate immunogenicity and, occasionally, trigger cutaneous hypersensitivity reactions.^6^, ^7^

Contrary to local reactions, vaccine exanthema typically occurs 1‒2 weeks after vaccination, similarly to viral exanthema, due to antigen‐induced antiviral immune activation.^8^

In our series, cutaneous manifestations following Butantan-DV vaccination were maculopapular and self-limited, with onset approximately one week after immunization, and a favorable outcome. The rash and the clinical picture of the Butantan-DV differ from those of dengue fever infection, chikungunya, or Zika, which is relevant in endemic areas. Zika typically produces an early-onset, centrifugally spreading pruritic maculopapular eruption, often accompanied by non-purulent conjunctivitis. Dengue exanthema usually appears later in the course of illness and may present as a morbilliform or scarlatiniform rash, sometimes displaying the classic pattern of “white islands in a sea of red”, and occasionally associated with petechiae. In contrast, chikungunya generally causes a more intense maculopapular eruption, frequently associated with marked arthralgia and sometimes followed by post-inflammatory hyperpigmentation, particularly involving the face or nasal region. Dermatoscopically, Zika lesions typically show an erythematous background with diffusely distributed dotted vessels, occasionally accompanied by very fine superficial scaling. Dengue lesions may demonstrate a mottled erythematous background with dotted or short linear vessels and scattered purpuric dots. In chikungunya, dermatoscopy often reveals diffuse erythema with dotted vessels and subtle perifollicular accentuation.^9^, ^10^, ^11^, ^12^

All 37,000 vaccinated inhabitants were instructed to seek medical care if they experienced any adverse event following vaccination, regardless of its specificity. Notwithstanding, this series was not designed to estimate adverse-event incidence; rather, it aimed to clinically characterize the rash pattern and its associated symptoms. Notably, in highly admixed populations, the prevalence of such eruptions may be underreported, as they are often oligosymptomatic, and the subtle maculopapular lesions may be underecognized in individuals with skin of color.

Recognition of these manifestations is particularly relevant for healthcare personnel in the context of large-scale vaccination campaigns. Familiarity with benign post-vaccination eruptions may assist in diagnosis, optimize patient counseling, and prevent unnecessary diagnostic investigations or therapeutic interventions. Further studies assessing histopathologic features and PCR detection of the attenuated vaccine virus in the lesions are warranted to better elucidate pathogenesis.

In conclusion, Butantan-DV vaccination may be associated with a delayed, self-limited maculopapular exanthem with a benign clinical course. Recognition of this pattern is relevant in endemic settings to avoid misdiagnosis and reinforce vaccine safety.

## Authors' contributions

Thays Carvalho: Study conception and design; data collection; data analysis and interpretation; manuscript drafting; critical literature review; critical manuscript revision; approval of the final version of the manuscript.

Aluisio Neto: Study conception and design; data collection; data analysis and interpretation; manuscript drafting; critical literature review; critical manuscript revision; approval of the final version of the manuscript.

Izabelle Bonini: Study conception and design; data collection; data analysis and interpretation; manuscript drafting; critical literature review; critical manuscript revision; approval of the final version of the manuscript.

Marilia Jorge: Study conception and design; data analysis and interpretation; manuscript drafting; critical literature review; critical manuscript revision; approval of the final version of the manuscript.

Marianna Oliveira: Study conception and design; data analysis and interpretation; manuscript drafting; critical literature review; critical manuscript revision; approval of the final version of the manuscript.

Hélio Miot: Study conception and design; data analysis and interpretation; manuscript drafting; critical literature review; critical manuscript revision; approval of the final version of the manuscript.

## Declaration of Generative AI and AI-assisted technologies in the writing process

During the preparation of this work, the authors used ChatGPT 5.2 to assist with English-language editing. After using this tool, the authors reviewed and edited the content as needed and take full responsibility for the content of the published article.

## Financial support

10.13039/501100003593CNPq (306358/2022-0) – Hélio Miot is a researcher from CNPq.

## Research data availability

Does not apply.

## Conflicts of interest

None declared.
